# Electrically Tunable Flexible Circularly Polarized Laser with Ultrahigh Asymmetry Factor

**DOI:** 10.1021/acsnano.5c13435

**Published:** 2025-10-14

**Authors:** Guodan Wei, Rui Duan, Yuan Wang, Tairan Yang, Tianhua Ren, Junzi Li, Yanyan Cui, Tesen Zhang, Handong Sun

**Affiliations:** Institute of Applied Physics and Materials Engineering, 59193 University of Macau, Macao SAR 999078, China

**Keywords:** circularly polarized laser, electrically tunable, high asymmetry factor, flexible soft photonic device, chiral light sources

## Abstract

Circularly polarized (CP) laser emission delivers considerable promise for future photonic applications of chiral light sources and investigation of chiral light–matter interactions. However, the low asymmetry factor (*g*_lum_) and lack of effective tuning have significantly hindered the development of such chiral light sources. Herein, multicolor (blue, green, and red) flexible CP lasers are demonstrated based on dye-doped cholesteric liquid crystal (CLC) microdroplets embedded in a polydimethylsiloxane (PDMS) elastomer. The CLC microdroplets, characterized by their left- or right-handed helical superstructures, serve as chiral cavities, facilitating the realization of CP lasing. By integrating chiral coating, CP lasing with opposite handedness is further effectively separated, notably enhancing the circular polarization degree and enabling an ultrahigh asymmetry factor (*g*_lum_ = 1.72). Importantly, these flexible CP lasers exhibit both electrically and mechanically tunable emission, demonstrating excellent wavelength tunability. In addition, the applied electric field allows dynamic control over the laser emission intensity, enabling fully reversible on/off switching. This work represents an important step forward to the development of high-performance chiral light sources with facile tunability and offers valuable insights for future chiroptical device design.

## Graphical abstract

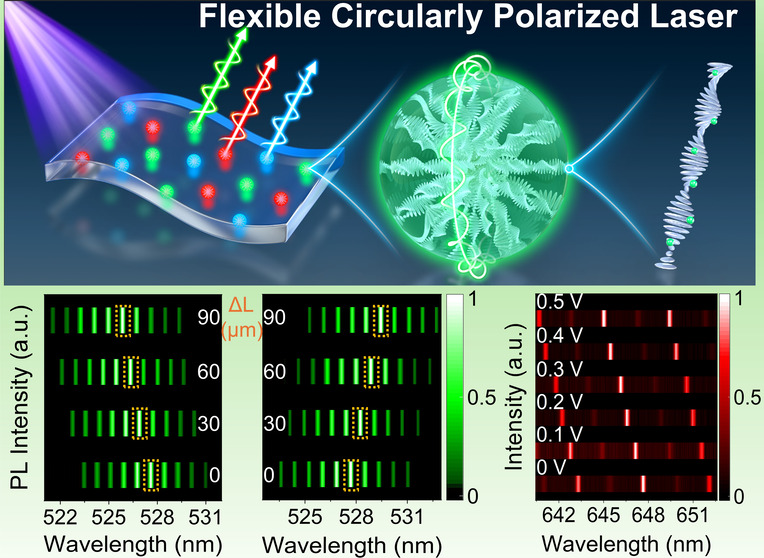

## Introduction

Circularly polarized (CP) light, characterized by its intrinsic chirality and controllable spin angular momentum, has emerged as a chiral light source for chirality-related applications, including 3D displays, information encryption, spin optical communication, and enantioselective photopolymerization.^1–5^ However, the incoherent broadband emission of traditional CP light typically results in poor spectral resolving power and inadequate color rendition, which largely hampers its application potential.^6,7^ Circularly polarized (CP) lasers, which deliver intense coherent CP light emission with an ultranarrow line width, have sparked a revolution in the field of chiroptics. Recently, CP lasers have been demonstrated in different resonant cavities, including organic microcrystals,^8–11^ cellulose nanocrystal cavities,^12^ chiral distributed Bragg reflectors (DBRs),^13^ chiral metasurface coupled cavities,^14–17^ and asymmetric or anisotropic cavities exploiting spin-split electronic bands and chiral photonic modes.^6,18,19^ While impressive demonstrations exist, the development of CP lasers still faces significant challenges, primarily because they pose considerable technical difficulties in simultaneously achieving a high degree of circular polarization, mechanical flexibility, dual wavelength tunability, and controllable output intensity, requiring exploiting novel microlasers with tailorable CP lasing properties.

To address these intertwined challenges in CP lasers, soft matter systems combining chiral photonics structure with tunability, flexibility, and stimulus responsiveness are highly favored.^20,21^ Cholesteric liquid crystals (CLCs), a canonical soft matter, can self-assemble into left-/right-handed helical superstructures that form one-dimensional photonic bandgaps (PBG) for selective reflection of CP light matching the helix handedness.^22,23^ Capitalizing on self-assembled helical superstructures, CLCs have been extensively explored as mirrorless resonant cavities for microlasers.^24–30^ Furthermore, the softness and fluidity of CLCs, as well as their excellent processability, enable the fabrication of flexible photonic devices with complex 3D helical superstructures, eliminating the need for conventional solid-state lithography techniques.^31^ Crucially, the liquid crystal molecular arrangement is sensitive to temperature and electric or magnetic fields, which permits precisely tuned emission output characteristics including wavelength, intensity, and polarization state.^32–34^ All these distinctive features make CLCs a promising candidate for the fabrication of CP lasers and have yielded promising results, including 3D CP laser display,^7^ thermoresponsive CP microlaser arrays,^35^ and droplet omnidirectional CP laser.^36,37^ The emission of these CP lasers has been effectively tuned by individual external stimuli, such as electrical,^38^ mechanical,^39^ or thermal^35^ in previous work. However, achieving CP lasers with high asymmetry factors that feature dual tunability—particularly with simultaneous electrical and mechanical tuning—remains a challenge, which hinders the development of CP lasers as next-generation chiral light sources.

Herein, we pay our attention to addressing the tuning issues in addition to demonstrating high-quality CP lasers with high asymmetric factors. We propose a multicolor, electrically tunable, flexible CP laser that leverages the unique advantages of CLCs. The CLCs were self-assembled into microdroplets with a periodic helical superstructure and embedded in a flexible polydimethylsiloxane (PDMS) elastomer. The CP lasers were achieved by doping gain dyes into CLC microdroplets with a helical superstructure that serve as chiral resonant cavities, realizing CP lasing with an asymmetric factor (*g*_lum_) of approximately 1. By integrating chiral coating on the surface of the PDMS elastomer, CP lasing emissions with opposite handedness were further effectively separated, notably enhancing the circular polarization degree and enabling an ultrahigh asymmetric factor (*g*_lum_ = 1.72). In addition, thanks to the good elasticity of PDMS, our CP laser is flexible and deformable, which enables us to achieve a strain-tuned CP lasing wavelength by applying mechanical force to stretch or bend the device. Furthermore, the lasing emission-tuned efficiently and precisely by an external electric field was demonstrated. The lasing emission wavelength can be tuned at low voltages (0.5 V), while the laser emission output can be controlled at high voltages (6 V). We believe that this study signifies the development of CP lasers and provides valuable insights into next-generation chiral light sources and chiroptical devices.

## Results and Discussion

### Fabrication and Characterization of Flexible CP Lasers

Multicolor flexible CP lasers were fabricated by embedding blue-, green-, and red-emissive CLC microdroplets into the PDMS elastomer, as schematically illustrated in Figure 1a. First, the helical ordering of liquid crystal molecules can be induced through introducing chiral dopants S5011 and R5011 (Figure S1) into nematic liquid crystals 5CB (Figure S2), where the molecular direction rotates azimuthally to obtain CLCs with left-handed and right-handed helical superstructures (Figure S3). To fabricate flexible CP lasers, three organic dyes were used, namely, 1,4-bis­(2-methylstyryl)­benzene (Bis-MSB), Coumarin 540A (C-540A), and Nile Red (NR) (Figure S4), which were selected as gain medium and incorporated into CLCs, which have photoluminescence (PL) emission in blue, green, and red wavebands, respectively (Figure 1h). Then, dye-doped CLCs were mixed with PDMS (10:1 mass ratio of liquid silicon base and curing agent) and spontaneously formed smooth spherical microdroplets due to their immiscibility with the PDMS matrix. After that, the CLC microdroplets/PDMS mixture was placed at room temperature to cure for 48 h to obtain flexible CP lasers.

**Figure 1:**
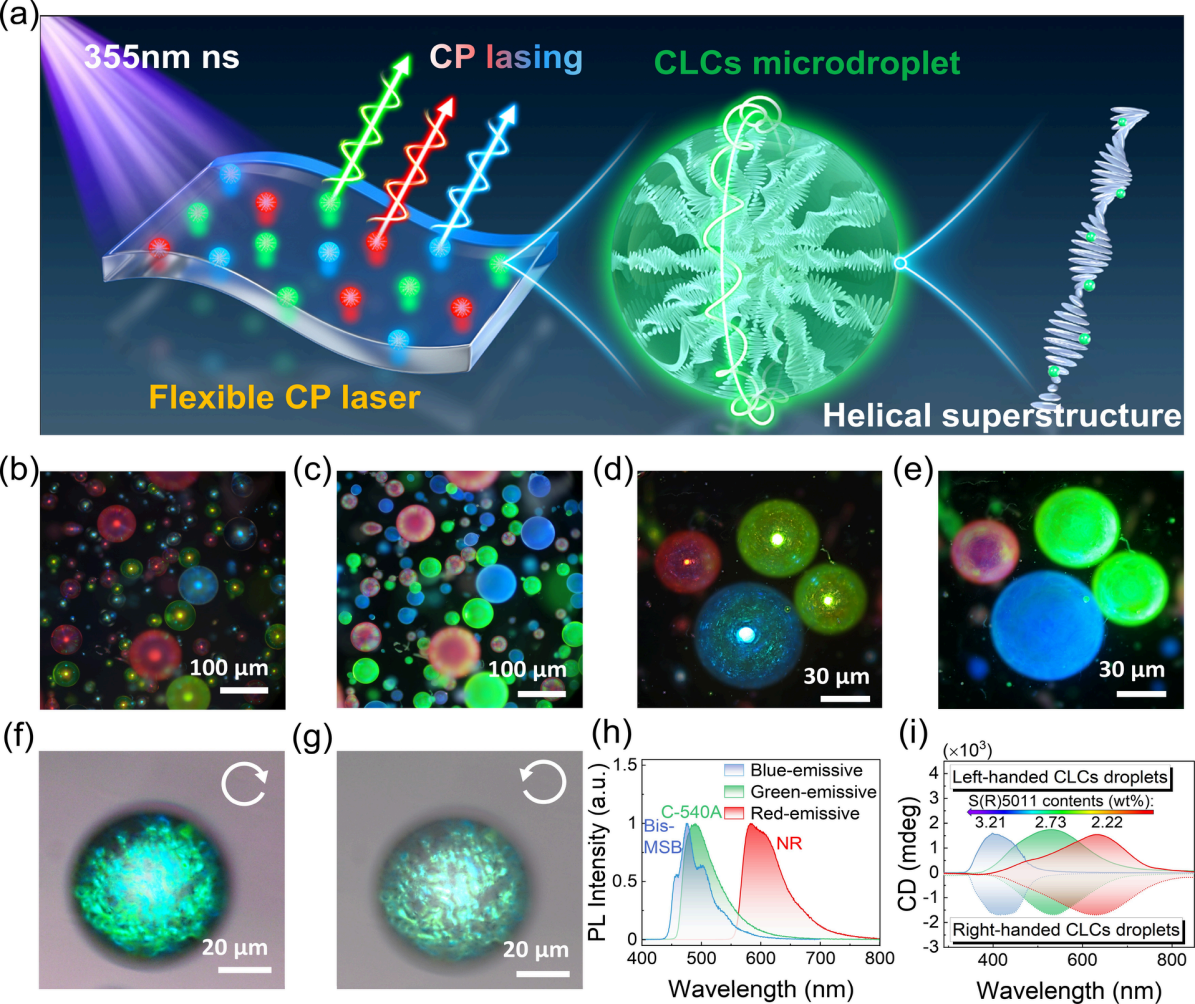
Fabrication and characterization of flexible CP lasers. (a) Schematic diagram of multicolor (blue, green, red) flexible CP lasers with dye-doped CLC microdroplets embedded in a PDMS elastomer, pumped by a nanosecond pulse of 355 nm. CLCs self-assemble to form microdroplets with complex 3D helical superstructures without conventional solid-state lithography techniques, serving as chiral cavities for CP lasing. (b–e) POM images show the presence of helical ordering of the liquid crystal molecules in CLC microdroplets, as well as the phase separation between the anisotropic CLC domains and the surrounding isotropic PDMS matrix. The fluorescent photographs indicate that the gain dyes are uniformly dispersed inside the microdroplets with no leakage into the PDMS matrix. (f, g) Microscope photographs of CLC microdroplets with left-handed helical superstructures taken using left-handed (f) and right-handed (g) circularly polarized filters, respectively. (h) PL spectra of blue-, green-, and red-emissive CLC microdroplets doped with different gain dyes. (i) CD spectra of CLC microdroplets containing different contents of chiral dopants.

The micrographs of the interior microscopic details of a typical CP laser taken using polarized optical microscopy (POM) are shown in Figure 1b–e. The CLCs form polydisperse spherical microdroplets with sizes in the range of 10*–*150 μm after PDMS solidification (Figure S5), confirming that the PDMS curing process does not cause deformation of CLCs microdroplets (Figure 1b,c). In Figure 1b, it is worth noting that some of the microdroplets are blurred, since they are located outside the focal plane as a result of the three-dimensional structure of our CP lasers. Under 365 nm UV light, it can be observed that the background around the microdroplet is black in the entire field of view, indicating that the gain dye has not leaked into the surrounding PDMS matrix (Figure 1c). The striking structural colors and bright spots that appear at the center of the microdroplets in reflection-mode POM originate from the Bragg reflection of periodic helical superstructures inside CLCs. The black background confirms a clear phase separation between the anisotropic CLC domains and the surrounding isotropic PDMS matrix (Figure 1d).^32^ The transmission-mode POM of CLC microdroplets shows the well-known Maltese cross when observed between crossed polarizers, which confirms the radial growth of the helical structure (Figure S6).^40^ Fluorescence photographs show these uniformly luminescent blue-, green-, and red-emissive microdroplets, indicating that the gain dyes are uniformly dispersed inside without aggregation occurring (Figure 1e), and the PL spectra are depicted in Figure 1h. The reflection color of CLC microdroplets arises from the Bragg reflection brought by their periodic helical superstructures.^34^ When circular polarized filters are used to allow the same-handedness CP light to pass and prohibit the opposite-handedness CP light, the CLC microdroplet with a left-handed helical superstructure that reflect left-handed CP light shows color saturation under the left-handed filter (Figure 1f) and dullness under the right-handed filter (Figure 1g), proving the excellent circular polarization properties of fabricated CLC microdroplets. The high circular dichroism (CD) intensities shown in Figure 1i further demonstrate the strong chirality of CLC microdroplets, supporting their feasibility as chiral cavities for CP lasers with opposite handedness. Additionally, we further adjusted the PBG across the entire visible-light range by precisely controlling the content of the chiral dopants to match the emission band of three gain dyes, ensuring that our CP laser has a high circular polarization degree (Figure 1i and Figure S3). Furthermore, the absorption spectrum indicates the coupling of the PBG effect of the CLCs and the inherent molecular absorption of the dyes, which establishes a favorable light–matter interaction to provide effective feedback for CP lasing, as depicted in Figure S7.

### Lasing from Multicolor CLC Microdroplet Microlasers

The lasing characteristics of dye-doped CLC microdroplets were investigated by a custom-built microphotoluminescence (μ-PL) system utilizing a Q-switched Nd:YAG pulsed laser for pumping (excitation: 355 nm, repetition rate: 20 Hz, pulse duration: 5 ns), as shown in Figure S8. Figure 2a–c plots the integrated PL intensity and full width at half-maximum (fwhm) of emission peaks as a function of the pump fluence. Below the lasing threshold, CLC microdroplets exhibit weak PL intensity and a broad fwhm, characteristic of spontaneous emission. Upon reaching the threshold fluence, the PL intensity increases rapidly and fwhm decreases sharply with the increasing pump pulse fluence. This simultaneous observation of a threshold behavior, a nonlinear increase in intensity, and spectral narrowing confirms the onset of lasing action.^41^ It should be noted that, using nanosecond laser pumping, the lasing thresholds for blue-, green- and red-emissive CLC microdroplets were determined to be as low as ∼10.44, ∼7.74, and ∼7.33 μJ cm^–2^, respectively, as illustrated in Figure 2a, Figure 2b, and Figure 2c, respectively, which are lower than that of most other CP lasers.^6,8,10,35,36^ When pump fluences exceeded their thresholds, the PL spectra developed as a set of sharp lasing spikes of blue, green, and red centered at ∼457.3, ∼527.5, and ∼640.2 nm, respectively, as depicted in Figure 2d–f. For red-emissive CLC microdroplets (diameter = 58 μm), the line width of the individual modes narrowed to 0.09 nm, corresponding to a lasing *Q*-factor (*Q* = λ/Δλ, where λ and Δλ are the lasing emission wavelength and peak line width, respectively) exceeding 7000 (Figure 2f), validating the high quality of the CLC resonant cavity. The laser peak line widths of both blue-emissive microdroplets (diameter = 62 μm) and green-emissive microdroplets (diameter = 60 μm) are less than 0.2 nm, and the laser *Q*-factors exceed 2800 and 4700, respectively. The insets in Figure 2d–f show bright ring-shaped circles at the outer boundaries of CLC microdroplets, indicating the formation of whispering gallery mode (WGM) resonances. Moreover, Figure S9 depicts a collection of lasing spectra from CLC microdroplets with different diameters. The inverse proportional dependence between free spectral range (FSR) and diameter (*D*) clearly indicates the WGM characteristic of CLC microdroplet microlasers.^42,43^

**Figure 2:**
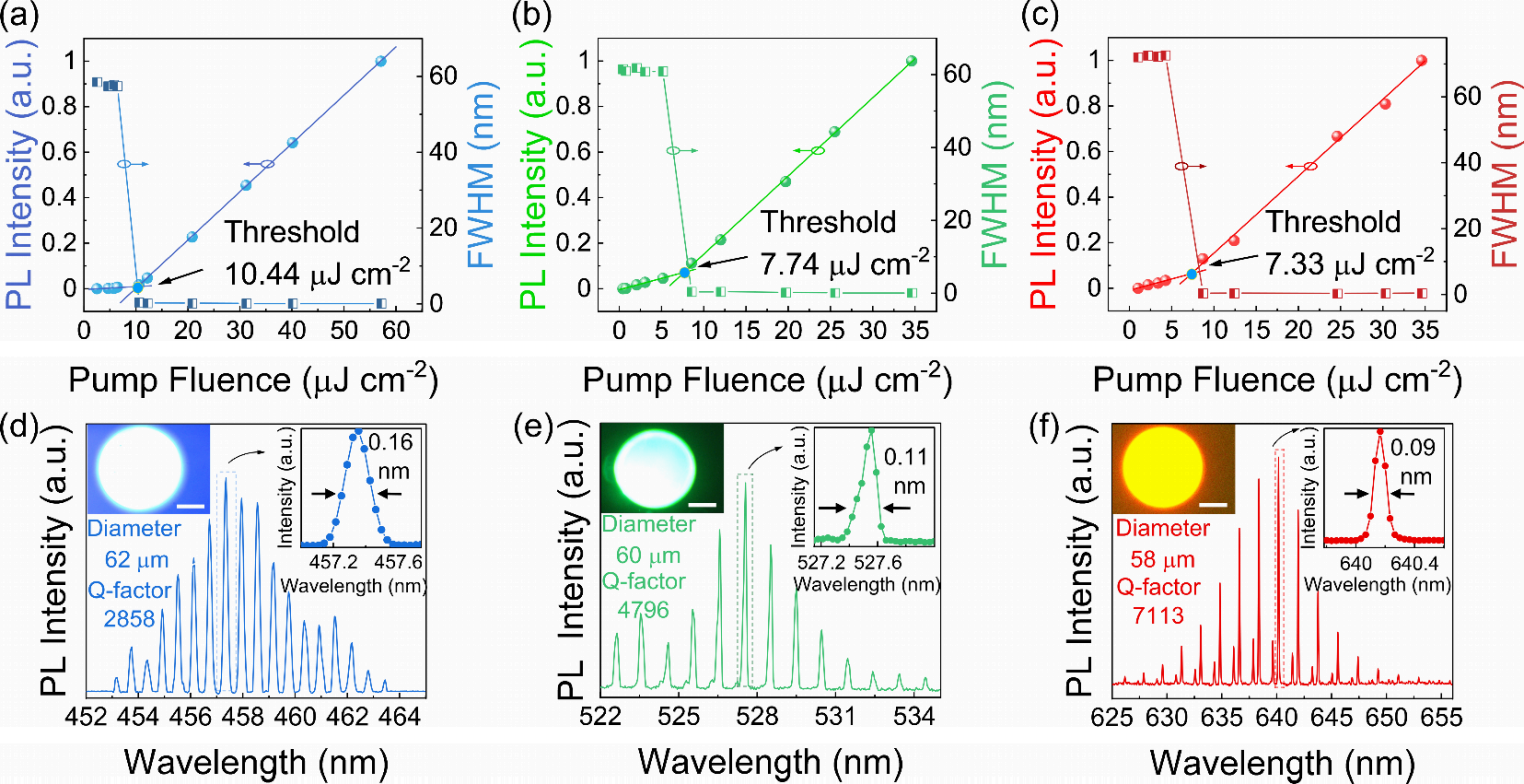
Lasing measurements of multicolor CLC microdroplet microlasers. (a–c) Evolution of the normalized PL intensity and fwhm of emission peaks as a function of different pump fluences for blue-emissive (a), green-emissive (b), and red-emissive (c) CLC microdroplets, respectively. (d–f) Lasing spectra with different emissive wavebands from blue-emissive (d), green-emissive (e), and red-emissive (f) CLC microdroplet microlaser, respectively. The left insets show the PL images of the CLC microdroplet microlasers, the diameters of the blue-, green-, and red-emissive droplets are 62, 60, and 58 μm, respectively. The right insets show the line width of the lasing peak.

### Circular Polarization Characteristic of CP Lasers

To characterize the circular polarization state from CP lasers, we first used a rotating analyzer where the intensity of the transmitted light passing through a quarter-wave plate (QWP) is recorded (Figure S10). The QWP can convert the CP light from CP lasers into a linearly polarized light, which is detected by a rotating linear polarizer (0–360°). As illuminated in Figure 3a–c, the left-handed CP lasing (LCP) and right-handed lasing (RCP) emission emitted by CLC microdroplet CP lasers were converted to linear polarized light with an orthogonal polarization plane, corresponding to +45 and −45° angular offsets relative to the long axis of QWP, respectively, showing excellent circular polarization characteristic of emission from CP lasers. Previous studies have shown that liquid crystal molecules tend to be radial anchoring in PDMS.^44^ Inside CLC microdroplets, liquid crystal molecules tend to form helical superstructures at the boundaries due to the anchoring effect of PDMS. Thanks to the PBG effect brought by the helical superstructure, the propagation of CP light with the same handedness as that of CLCs is forbidden, whereas CP light with opposite handedness is transmitted.^1,7^ When green-emissive CLC microdroplets with a right-handed superstructure are pumped by a nanosecond laser, light circulates through the helical superstructure along the equator of the microdroplet (Figure 1a), and RCP light is suppressed while LCP light is allowed, resulting in an LCP lasing emission and vice versa (Figure 3e). The blue- and green-emissive CP lasing emissions with opposite handedness were also successfully obtained from the corresponding CLC microdroplets, as depicted in Figure 3d and Figure 3f, respectively. A critical parameter used to quantify the circular polarization degree of CP lasing is the asymmetry factor (*g*_lum_), which is defined mathematically as *g*_lum_ = 2 × (*I*_L_ – *I*_R_)/(*I*_L_ + *I*_R_), where *I*_L_ and *I*_R_ denote the emission intensities of LCP lasing and RCP lasing, respectively.^45^ The *g*_lum_ values of blue-, green-, and red-emissive CP lasers were calculated, and the |*g*_lum_| values were determined to be as high as ∼0.89, ∼1.06, and ∼0.92, respectively, as plotted in Figure 3g–i. This result highlights the significant potential of CLC microdroplets as excellent chiral resonant cavities.

**Figure 3:**
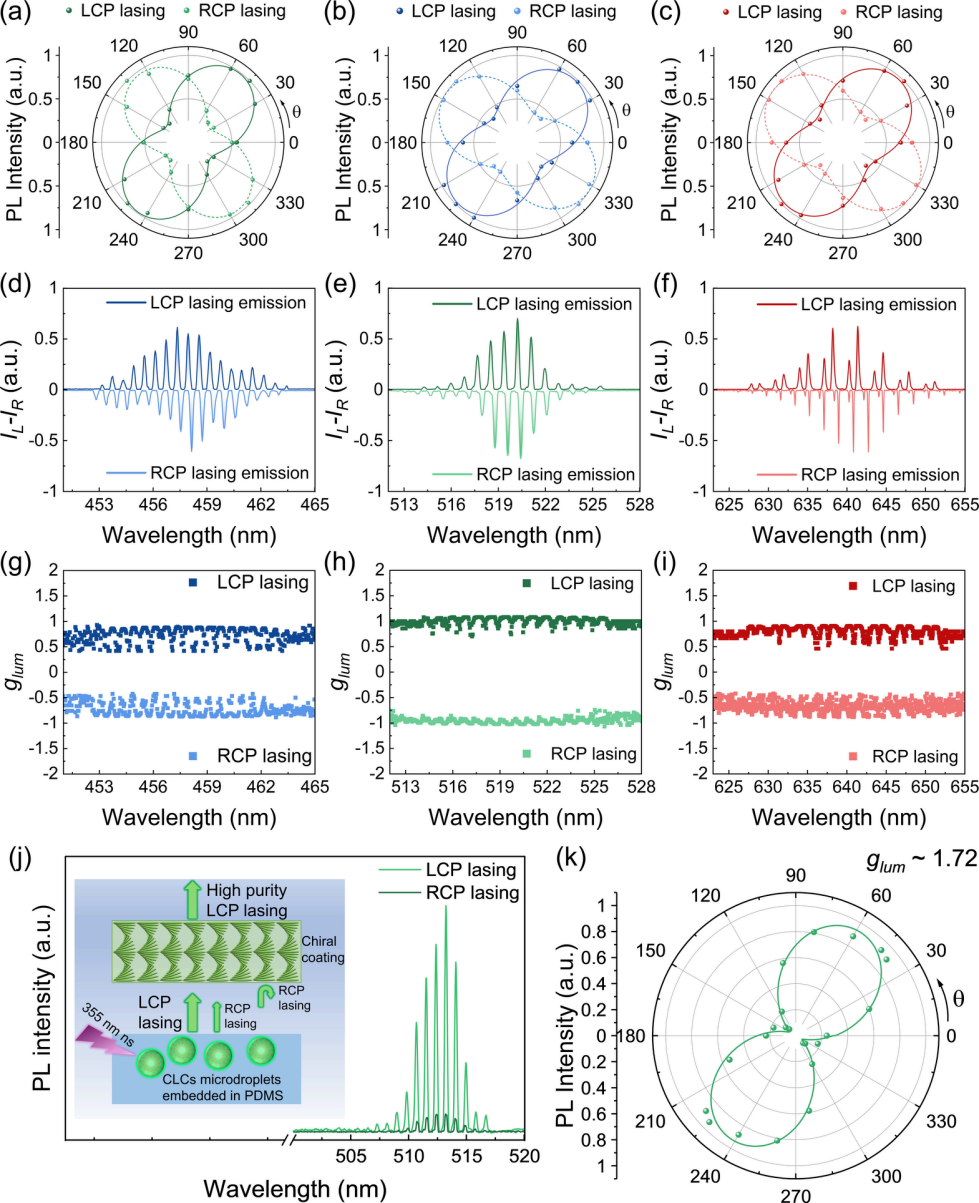
Circular polarization characterization of CP lasers. (a–c) Polarization angle-dependent emission intensity of the left-/right-handed blue-emissive (a), green-emissive (b), red-emissive (c) CLC microdroplet microlasers after passing through a quarter-wave plate, where θ refers to the polarization angle. (d–f) CP lasing emission from left-/right-handed blue-emissive (d), green-emissive (e), and red-emissive (f) CLC microdroplet microlasers, respectively. (g–i) *g*_lum_ spectra versus wavelength for LCP lasing and RCP lasing emission of blue-emissive (g), green-emissive (h), and red-emissive (i) CLC droplet microlasers, respectively. (j) High-purity LCP lasing from chiral coating-integrated CP laser. The inset shows the mechanisms of chiral coating efficiently separating LCP lasing and RCP lasing. The RCP lasing was selectively reflected to prevent propagation, while the LCP lasing was transmitted, thereby enabling high-purity LCP lasing output. (k) Lasing emission intensity as a function of the polarization angle from the chiral coating-integrated CP laser.

Amplifying the degree of circular polarization of CP lasers is of great significance for broadening the application of chiral light sources,^46^ which requires a *g*_lum_ value approach to the extreme of 2 to achieve high-purity CP lasing emission. We aim to achieve high-purity CP lasing emission by integrating a chiral coating on a CP laser device. Chiral coatings are integrated into the surface of PDMS elastomer by the spin-coating method (see detailed fabrication protocol available in the Experimental Section in the Supporting Information). For example, a right-handed chiral coating is integrated onto the top surface of a CP laser that emits LCP lasing, as schematically illustrated in Figure S11. The cross-sectional SEM image shows that the chiral coating with a thickness of ∼45 μm is tightly attached to the PDMS elastomer (Figure S12). Figure S13 shows the POM image of the chiral coating, where the presence of a typical oily streak texture serves as evidence for the formation of chiral structures. The right-handed chiral coating transmits LCP light while suppressing RCP light and can be used as a CP filter (Figure S14). When the CP laser device is pumped, the laser emission consists predominantly of LCP lasing and a small amount of RCP lasing, with the LCP lasing propagated through the chiral coating, whereas the RCP lasing is forbidden by selective Bragg reflection, as schematically depicted in the inset of Figure 3j. After integration of the chiral coating, the RCP lasing intensity decreased by 72.7% compared with the device without the coating (Figure S15). The chiral coating efficiently separates LCP lasing and RCP lasing, enabling a high-purity LCP lasing emission output (Figure 3j). Figure 3k shows the polarization angle-dependent emission intensity of laser emission, indicating the outstanding circular polarization degree of LCP lasing, with a *g*_lum_ value calculated to be approximately 1.72, which is comparable to that of high-performance CP lasers reported recently (Table S1). The *g*_lum_ value comparison of the control experiment also confirms that the chiral coating enhances the polarization selectivity provided by the chiral cavity (Figure S16). Additionally, the *g*_lum_ values measured on three spots (microdroplets) across the same device and on three independently prepared devices show excellent consistency, showing that our CP lasers are robust and reliable (Figure S17andTable S2). The ultrahigh asymmetry factor provides an excellent foundation for promoting the application of CP lasers in the field of chiral light sources.

### Electrical Tunability of Flexible CP Lasers

Electrically precise tuning over CP light, especially wavelength and intensity, is critical for technologies requiring on-demand chiral light sources, such as tunable chiral photonics, reconfigurable quantum optical systems, and display technologies.^47,48^ We aim to apply an external electric field to the proposed CP lasers to achieve electrically tunable lasing emission. Before that, the mechanical tunability of the flexible CP laser was demonstrated to verify the feasibility of electrically driven tuning. As schematically shown in Figure 4a, the two ends of the chiral coating-integrated CP laser are anchored to a movable substrate. The chiral coating-integrated CP laser, measuring ∼1 cm in length and ∼1 mm in thickness, undergoes spontaneous stretch or bend deformation in response to bidirectional substrate movement (Δ*L*), enabled by the elasticity of PDMS and external mechanical actuation, confirming that our CP laser device is flexible. Deformation increases with increasing Δ*L*, and real-time spectral monitoring during mechanical deformation reveals mechanical-strain-dependent lasing wavelength tuning (Figure 4b). Interestingly, stretching the CP laser device induced systematic blueshifts of the lasing wavelength (left column inFigure [Fig fig4]b), while compressive bending produced progressive redshifts (right column inFigure [Fig fig4]b). The shift of the laser peak wavelength is attributed to the change in the refractive index of PDMS.^49^ The refractive index of PDMS is very sensitive to force.^50^ Tensile stress and compressive strain, respectively, cause its refractive index to decrease or increase, resulting in a blue shift or red shift of the laser peak wavelength.^49^ Additionally, the *g*_lum_ value decreases slightly but remains high at 1.34 and 1.41 for stretched or bent CP laser (Δ*L* = 90 μm), respectively, which indicates that the circular polarization characteristics of CP lasers remain during deformation (Figure S18). This result demonstrates the high sensitivity of the laser peak to the refractive index change, laying the excellent foundation for electrically driven tunable CP lasers.

**Figure 4:**
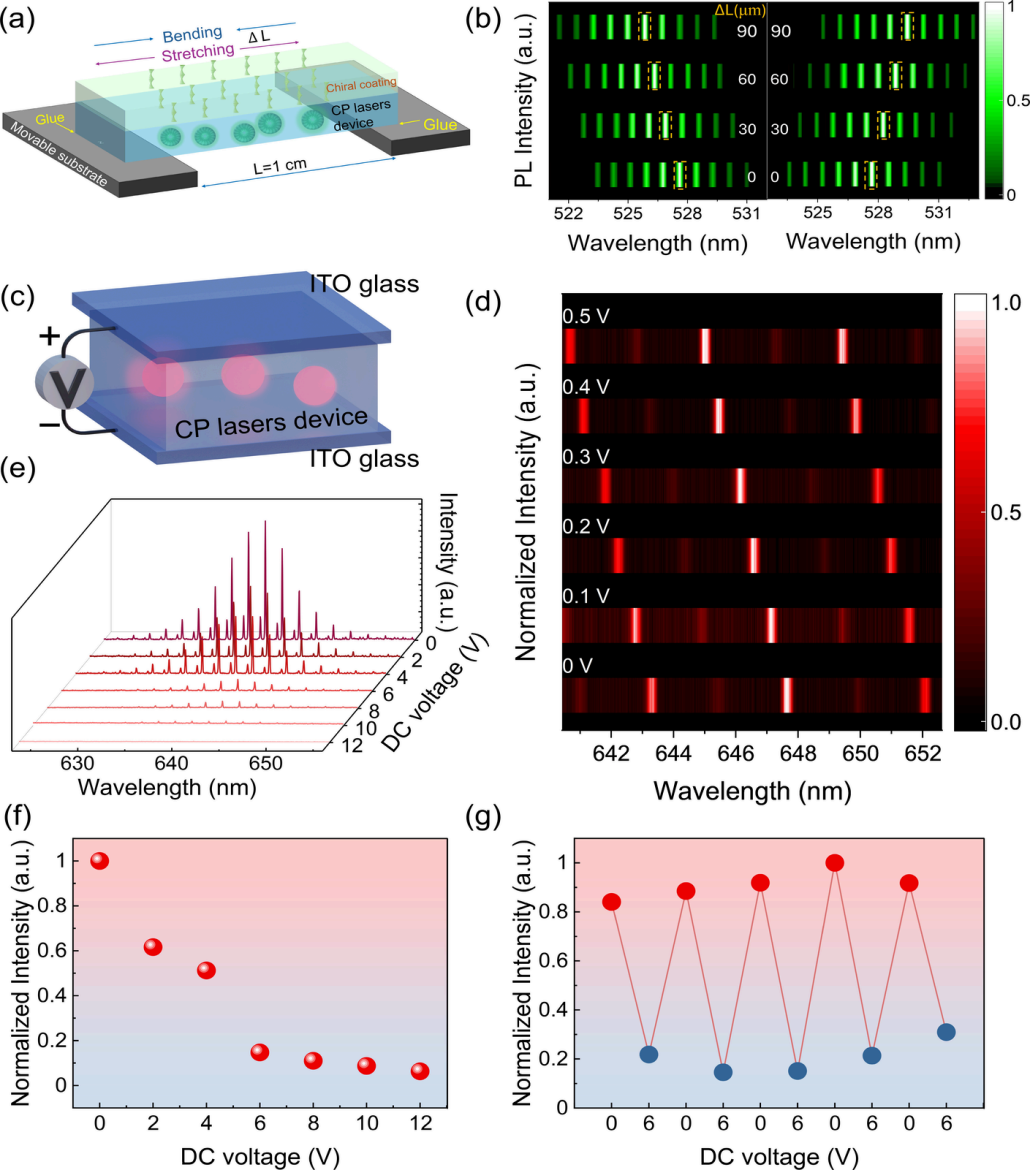
Electrical tunability of flexible CP lasers. (a) Schematic diagram shows the stretching or bending of the flexible CP laser. (b) Mechanical-strain–dependent shift of lasing emission of the CP laser under different degrees of stretching (left column) or bending (right column). (c) Schematic diagram of an electrically tunable CP laser device. (d) Electric-field–dependent shift of the lasing emission of CP lasers under 0–0.5 V electric field. (e) Evolution of the lasing emission in the range of 0–12 V applied voltage. (f) Laser emission output intensity as a function of applied voltage. (g) Lasing emission intensity for consecutive voltage cycles of 0–6 V applied to the device.

Liquid crystal molecules are electrically responsive, with their refractive index changing when an electric field is applied, which makes it possible to achieve an electrically driven tunable lasing emission. We fabricate an electrically tunable CP laser device with a sandwich structure (see detailed fabrication protocol available in the Experimental Section in the Supporting Information), which is a CP laser sandwiched between two pieces of ITO glass, with an electric field supplied through a direct current (DC) power source (Figure 4c andFigure S19). Figure 4d illustrates the electric-field–dependent shift of the lasing emission over a voltage range of 0–0.5 V, with the emission wavelength electrically tuned to a range of ∼2.7 nm. This electrical tuning range is sufficient for highly sensitive sensing applications that rely on tracking minute resonance shifts and offers a critical advantage for the future development of high-resolution integrated sensor devices. Owing to the positive dielectric anisotropy (Δε > 0) of the 5CB liquid crystal molecules, the orientation distribution of the long axis of the liquid crystal molecules tends to be parallel to the electric field,^33,44^ which leads to a decrease in the refractive index of the CLC microdroplet cavity, consequently to a blue shift of the lasing emission wavelengths. This result demonstrates the excellent electrical tunability of the proposed CP lasers, providing a solid foundation for efficient and precise operation in future applications. Additionally, the intensity of emission can also be tuned by the electric field under a relatively high voltage. Under a sufficiently strong electric field, both the liquid crystal molecules (Δε > 0) and doped gain dyes align predominantly along the field direction, namely, perpendicular to the polarization direction of the nanosecond pump laser. This configuration results in a significant decrease in the excitation photon absorption by the gain medium and, consequently, the intensity of lasing emission.^32^ As depicted in Figure 4e, when the applied voltage exceeds 2 V, the laser emission intensity shows a directed change, manifesting as a decrease with increasing voltage. When the voltage reaches 6 V, the variation in the emission intensity can be approximately 85% of the maximum value (Figure 4f). This allows us to easily realize reversible control of the laser output by switching on or off the applied electric field (Figure 4g). It is worth noting that the switching behavior demonstrated here under DC electric fields represents a quasi-static response. The switching kinetics, such as response times, are expected to be in the order of milliseconds to seconds, consistent with the reorientation dynamics of liquid crystal molecules.^51,52^ Overall, the proposed CP laser can provide precise electrical tunability of the lasing emission wavelength and intensity, and we believe that this electrically tunable flexible CP laser may drive the development of high-precision and high-performance chiral light sources.

## Conclusions

In summary, we have successfully demonstrated electrically tunable flexible CP lasers with an ultrahigh asymmetry factor. By exploiting CLC microdroplets with self-assembled helical superstructures as chiral cavities, our CP lasers realize multicolor (blue, green, red) CP lasing with opposite handedness. In addition, the integration of chiral coating onto the CP laser offers an exceptionally high degree of circular polarization, achieving an asymmetry factor as high as 1.72. More importantly, our flexible CP lasers exhibit dual tunability, both mechanical and electrical, enabling precise and effective wavelength modulation. Most notably, the application of an external electric field permits dynamic and reversible control of the lasing intensity, including complete on/off switching. The circular polarization properties of flexible CP lasers, combined with their responsivity to external signals, might pave the way for soft matter chiroptical photonic devices. Finally, these novel CP lasers may contribute to further understanding of chiral light–matter interactions and hold promise as next-generation chiral light sources for optical communications, information encryption, 3D displays, and diagnostic devices.

## Methods and Experimental Section

### Fabrication of CP Lasers

First, the nematic liquid crystal 5CB (*n*_o_ = 1.53, *n*_e_ = 1.71 at 589 nm) was mixed with different contents of chiral dopant (S5011 or R5011) to obtain cholesteric liquid crystals with different photonic band gaps, which exhibit blue, green, and red structural colors, respectively. Then, Bis-MSB (blue gain dye), C-540A (green gain dye), and NR (red gain dye) were doped into CLCs with tailored photonic band gaps. Next, the dye-doped CLCs were subjected to ultrasonic treatment for 10 min to achieve homogeneous dispersion. Subsequently, homogeneously dispersed dye-doped CLCs were mixed with PDMS (10:1 mass ratio of liquid silicon base and curing agent, refractive index after solidification: 1.42) by mechanical stirring and spontaneously formed smooth spherical microdroplets of sizes in the range of 10 μm–150 μm. Finally, dye-doped CLC microdroplets/PDMS mixtures were poured into a Petri dish and cured at room temperature for 48 h under a nitrogen atmosphere to obtain flexible CP lasers.

### Fabrication of Electrically Tunable CP Laser Devices

First, the CLC microdroplets/PDMS mixtures (20 μL) as an active layer were dropped on a quartz substrate (1 cm × 2 cm) coated with an indium–tin-oxide (ITO) layer, and a second piece of ITO-covered quartz glass was subsequently placed on top of the active layer. Prior to measurements, the samples were stored for 48 h at room temperature under a nitrogen atmosphere so that the PDMS could completely cross-link. The applied electric field was delivered by a direct current (DC) power source (DINGCE, DC1533D) through the ITO electrodes.

### Measurement of Lasing Spectral and Circular Polarization Characterization

Lasing spectra were acquired using a nanosecond pulsed laser (λ = 355 nm, pulse width = 5 ns, repetition rate = 20 Hz). Pump-intensity-dependent emission was collected with a fiber-coupled spectrometer and detected with a silicon-charge-coupled device (CCD). A USB 3.0 CMOS microscopy camera (model: YS2000) was used to capture spot size images, facilitating the calculation of spot area and subsequent determination of pump fluence from the measured intensities. To characterize the circular polarization properties of lasing emissions, a quarter-waveplate and a linear polarizer were placed in front of the fiber-coupled spectrometer, and the light signal that passed through the quarter-waveplate was collected by rotating the polarizer.

## Supplementary Material



## Notes

Device fabrication, measurement of steady-state photoluminescence (PL), transmission spectra, circular dichroism (CD) spectrum, scanning electron microscopy (SEM) characterization, and laser measurements (PDF)

## References

[1] Zhang M., Guo Q., Li Z., Zhou Y., Zhao S., Tong Z., Wang Y., Li G., Jin S., Zhu M., Zhuang T., Yu S. H. (2023). Processable circularly polarized
luminescence material enables flexible stereoscopic 3D imaging. Sci. Adv..

[2] Zhang X., Li L., Chen Y., Valenzuela C., Liu Y., Yang Y., Feng Y., Wang L., Feng W. (2024). Mechanically tunable
circularly polarized luminescence of liquid crystal-templated chiral
perovskite quantum dots. Angew. Chem., Int.
Ed..

[3] Song I., Ahn J., Ahn H., Lee S. H., Mei J., Kotov N. A., Oh J. H. (2023). Helical
polymers for dissymmetric circularly polarized light imaging. Nature.

[4] Han D., Yang X., Han J., Zhou J., Jiao T., Duan P. (2020). Sequentially amplified
circularly polarized ultraviolet luminescence
for enantioselective photopolymerization. Nat.
Commun..

[5] Chowdhury R., Preuss M. D., Cho H. H., Thompson J. J. P., Sen S., K. Baikie T., Ghosh P., Boeije Y., Chua X. W., Chang K. W., Guo E., van der Tol J., van den Bersselaar B.
W. L., Taddeucci A., Daub N., Dekker D. M., Keene S. T., Vantomme G., Ehrler B., Meskers S. C. J., Rao A., Monserrat B., Meijer E. W., Friend R. H. (2025). Circularly polarized electroluminescence
from chiral supramolecular semiconductor thin films. Science.

[6] Liang Q., Ma X., Long T., Yao J., Liao Q., Fu H. (2023). Circularly
Polarized Lasing from a Microcavity Filled with Achiral Single-Crystalline
Microribbons. Angew. Chem., Int. Ed..

[7] Zhan X., Xu F. F., Zhou Z., Yan Y., Yao J., Zhao Y. S. (2021). 3D laser displays based on circularly
polarized lasing
from cholesteric liquid crystal arrays. Adv.
Mater..

[8] Ren S., Liu Z. F., Li P., Liu H., Lu M., Wang K., Yao J., Dong H., Yang Q. Z., Zhao Y. S. (2025). Circularly polarized lasing from
helical superstructures
of chiral organic molecules. Angew. Chem., Int.
Ed..

[9] Ji S., Zhou Y., Xiong L., Liu X., Zhu T., Zhan X., Yan Y., Yao J., Wang K., Zhao Y. S. (2025). Nonreciprocal Circularly
Polarized Lasing from Organic
Achiral Microcrystals. J. Am. Chem. Soc..

[10] Ji S., Zeng M., Zhan X., Liu H., Zhou Y., Wang K., Yan Y., Yao J., Zhao Y. S. (2024). Exceptionally
high-g lum circularly polarized lasers empowered by strong 2D-Chiroptical
response in a host–guest supramolecular microcrystal. J. Am. Chem. Soc..

[11] Liu Z. F., Ren J., Li P., Niu L. Y., Liao Q., Zhang S., Yang Q. Z. (2023). Circularly
polarized laser emission from homochiral
superstructures based on achiral molecules with conformal flexibility. Angew. Chem., Int. Ed..

[12] Qu D., Archimi M., Camposeo A., Pisignano D., Zussman E. (2021). Circularly polarized laser with chiral nematic cellulose
nanocrystal cavity. ACS Nano.

[13] Wang C., Zhang W., Zhao H., Sun B., Zhao X., Luo D., Gao Y. (2025). High-Purity Circularly
Polarized Lasing From Chiral
VCSELs With Aligned Semiconductor Quantum Wells. Adv. Funct. Mater..

[14] Jia X., Kapraun J., Wang J., Qi J., Ji Y., Chang-Hasnain C. (2023). Metasurface reflector enables room-temperature circularly
polarized emission from VCSEL. Optica.

[15] Demenev A. A., Kulakovskii V. D., Schneider C., Brodbeck S., Kamp M., Höfling S., Lobanov S. V., Weiss T., Gippius N. A., Tikhodeev S. G. (2016). Circularly polarized lasing in chiral modulated semiconductor
microcavity with GaAs quantum wells. Appl. Phys.
Lett..

[16] Zhang X., Liu Y., Han J., Kivshar Y., Song Q. (2022). Chiral emission from
resonant metasurfaces. Science.

[17] Yuan Z., Huang S.-H., Qiao Z., Wu P. C., Chen Y.-C. (2023). Metasurface-tunable
lasing polarizations in a microcavity. Optica.

[18] Chen J.-Y., Wong T.-M., Chang C.-W., Dong C.-Y., Chen Y.-F. (2014). Self-polarized
spin-nanolasers. Nat. Nanotechnol..

[19] Duan X., Wang B., Rong K., Liu C.-l., Gorovoy V., Mukherjee S., Kleiner V., Koren E., Hasman E. (2023). Valley-addressable
monolayer lasing through spin-controlled Berry phase photonic cavities. Science.

[20] Ta V. D., Wang Y., Sun H. (2019). Microlasers
enabled by soft-matter
technology. Adv. Opt. Mater..

[21] Duan R., Thung Y. T., Zhang Z., Durmusoglu E. G., He Y., Xiao L., Lee C. X. X., Lew W. S., Zhang L., Li H., Yang J., Demir H. V., Sun H. (2024). Colloidal nanoplatelets-based
soft matter technology for photonic interconnected networks: Low-threshold
lasing and polygonal self-coupling microlasers. Laser Photonics Rev..

[22] Mitov M. (2012). Cholesteric
liquid crystals with a broad light reflection band. Adv. Mater..

[23] Zhou M., Lu H., Zhang X., Zhang Q., Xu M., Zhu J., Zhang G., Ding Y., Qiu L. (2019). Tuning helical twisting
power and photoisomerisation kinetics of axially chiral cyclic azobenzene
dopants in cholesteric liquid crystals. Liq.
Cryst..

[24] Furumi S. (2010). Recent progress
in chiral photonic band-gap liquid crystals for laser applications. Chem. Rec..

[25] Coles H., Morris S. (2010). Liquid-crystal lasers. Nat. Photonics.

[26] Wang C., Gong C., Zhang Y., Qiao Z., Yuan Z., Gong Y., Chang G.-E., Tu W.-C., Chen Y.-C. (2021). Programmable
rainbow-colored optofluidic fiber laser encoded with topologically
structured chiral droplets. ACS Nano.

[27] Finkelmann H., Kim S. T., Muñoz A., Palffy-Muhoray P., Taheri B. (2001). Tunable Mirrorless Lasing in Cholesteric Liquid Crystalline
Elastomers. Adv. Mater..

[28] Shi J., Ma C., Ren M., Xu M., Zhu J., Qiu L., Ding Y., Zhang J., Lu H. (2022). Stable and tunable
single-mode lasers based on cholesteric liquid crystal microdroplets. Appl. Opt..

[29] Lu H., Yang L., Xia L., Kong J., Xu M., Zhu J., Qiu L., Hu Z. (2021). Band-edge-enhanced tunable random
laser using a polymer-stabilised cholesteric liquid crystal. Liq. Cryst..

[30] Lu H. B., Xie X. Y., Xing J., Xu C., Wu Z. Q., Zhang G. B., Lv G. Q., Qiu L. Z. (2016). Wavelength-tuning
and band-broadening of a cholesteric liquid crystal induced by a cyclic
chiral azobenzene compound. Opt. Mater. Express.

[31] Papič M., Mur U., Zuhail K. P., Ravnik M., Muševič I., Humar M. (2021). Topological liquid crystal superstructures as structured light lasers. Proc. Natl. Acad. Sci. U. S. A..

[32] Adamow A., Szukalski A., Sznitko L., Persano L., Pisignano D., Camposeo A., Mysliwiec J. (2020). Electrically
controlled white laser
emission through liquid crystal/polymer multiphases. Light: Sci. Appl..

[33] Xiang J., Varanytsia A., Minkowski F., Paterson D. A., Storey J. M., Imrie C. T., Lavrentovich O. D., Palffy-Muhoray P. (2016). Electrically
tunable laser based on oblique heliconical cholesteric liquid crystal. Proc. Natl. Acad. Sci. U.S.A..

[34] Yuan C.-l., Huang W., Zheng Z.-g., Liu B., Bisoyi H. K., Li Y., Shen D., Lu Y., Li Q. (2019). Stimulated transformation
of soft helix among helicoidal, heliconical, and their inverse helices. Sci. Adv..

[35] Zhan X., Zhou Z., Zhou W., Yan Y., Yao J., Zhao Y. S. (2023). Wavelength-Tunable Circularly Polarized Laser Arrays
for Multidimensional Information Encryption. Adv. Opt. Mater..

[36] Humar M., Muševič I. (2010). 3D microlasers from self-assembled
cholesteric liquid-crystal microdroplets. Opt.
Express.

[37] Humar M. (2016). Liquid-crystal-droplet
optical microcavities. Liq. Cryst..

[38] Lu H., Wei C., Zhang Q., Xu M., Ding Y., Zhang G., Zhu J., Xie K., Zhang X., Hu Z. (2019). Wide tunable
laser based on electrically regulated bandwidth broadening in polymer-stabilized
cholesteric liquid crystal. Photonics Res..

[39] Shibaev P. V., Rivera P., Teter D., Marsico S., Sanzari M., Ramakrishnan V., Hanelt E. (2008). Color changing and lasing stretchable
cholesteric films. Opt. Express.

[40] Belmonte A., Bus T., Broer D. J., Schenning A. P. H. J. (2019). Patterned Full-Color Reflective Coatings
Based on Photonic Cholesteric Liquid-Crystalline Particles. ACS Appl. Mater. Interfaces.

[41] Duan R., Zhang Z., Xiao L., Zhao X., Thung Y. T., Ding L., Liu Z., Yang J., Ta V. D., Sun H. (2022). Ultralow-Threshold
and High-Quality Whispering-Gallery-Mode Lasing
from Colloidal Core/Hybrid-Shell Quantum Wells. Adv. Mater..

[42] Wang Y., Hu Y.-H., Wu J.-L., Tang J., Jiao Y.-F., Liang Y.-C., Wang H.-Y., Jiang L.-Y., Kuang L.-M., Xia K.-Y. (2025). Microcavity-based
parallel measurements of optical
power and wavelength. Appl. Phys. Rev..

[43] Thung Y. T., Duan R., Durmusoglu E. G., He Y., Xiao L., Lee C. X. X., Lew W. S., Zhang L., Demir H. V., Sun H. (2023). Ultrahigh Quality Microlasers from
Controlled Self-Assembly of Ultrathin
Colloidal Semiconductor Quantum Wells. Laser
Photonics Rev..

[44] Humar M., Ravnik M., Pajk S., Muševič I. (2009). Electrically
tunable liquid crystal optical microresonators. Nat. Photonics.

[45] Deng Y., Wang M., Zhuang Y., Liu S., Huang W., Zhao Q. (2021). Circularly polarized luminescence
from organic micro-/nano-structures. Light:
Sci. Appl..

[46] Sang Y., Han J., Zhao T., Duan P., Liu M. (2020). Circularly Polarized
Luminescence in Nanoassemblies: Generation, Amplification, and Application. Adv. Mater..

[47] Li Z., Lan R., Bao J., Hu W., Wang M., Zhang L., Yang H. (2022). Tunable Circularly Polarized Luminescence with a High Dissymmetry
Factor Emitted from Luminogen-Bonded and Electrically Controlled Polymer-Stabilized
Cholesteric Liquid Crystals. ACS Appl. Mater.
Interfaces.

[48] Lin W.-H., Wu P. C., Akbari H., Rossman G. R., Yeh N.-C., Atwater H. A. (2022). Electrically Tunable and Dramatically Enhanced Valley-Polarized
Emission of Monolayer WS2 at Room Temperature with Plasmonic Archimedes
Spiral Nanostructures. Adv. Mater..

[49] Chen R., Ta V. D., Sun H. (2014). Bending-Induced Bidirectional
Tuning
of Whispering Gallery Mode Lasing from Flexible Polymer Fibers. ACS Photonics.

[50] Markos C., Vlachos K., Kakarantzas G. (2010). Bending loss and thermo-optic effect
of a hybrid PDMS/silica photonic crystal fiber. Opt. Express.

[51] Lu H., Zhang J., Wang M., Song Z., Xiong X., Lin G., Zhang G., Wang X., Qiu L. (2014). Influence of Curing
Frequency on the Morphology and the Electro-Optical Property of Polymer-Stabilized
Cholesteric Textures. Mol. Cryst. Liq. Cryst..

[52] Mitsuishi M., Ito S., Yamamoto M., Fischer T., Knoll W. (1997). Time-resolved optical
waveguide study of the reorientation in a nematic liquid crystal under
applied electric field. J. Appl. Phys..

