# Supplementary material for: Electrically Tunable Flexible Circularly Polarized Laser with Ultrahigh Asymmetry Factor
Source: ACS Nano. 2025 Oct 14;19(42):37364–72. doi: 10.1021/acsnano.5c13435 (PMC12574215; doi:10.1021/acsnano.5c13435)
Supplement: Supplementary file 1 [file nn5c13435_si_001.pdf]

Supporting Information for

Electrically Tunable Flexible Circularly Polarized Laser with Ultra-High Asymmetry Factor

Guodan Wei[#], Rui Duan[#,], Yuan Wang, Tairan Yang, Tianhua Ren, Junzi Li, Yanyan Cui, Tesen Zhang, Handong Sun**

G.D. Wei, Dr. R. Duan, Y. Wang, T.R. Yang, Dr. T. H. Ren, Dr. J. Z. LI, Y.Y. Cui, Dr. T.S. Zhang, Prof. H. D. Sun

Institute of Applied Physics and Materials Engineering, University of Macau, Macao SAR 999078, China

*E-mail: ruiduan@um.edu.mo, hdsun@um.edu.mo

[#] These authors contributed equally to this work

Contents

Experimental Section S-3

S1. Materials.. S-3

S2. Fabrication of CP Lasers S-3

S3. Fabrication of Chiral Coating-Integrated CP Lasers S-4

S4. Fabrication of Electrically Tunable CP Laser Devices S-4

S5. Measurement of Steady-state Photoluminescence (PL), Transmission spectra, and Circular Dichroism (CD) spectrum. S-4

S6. Scanning Electron Microscope (SEM) Characterization and Polarized Optical Microscope (POM) Measurements S-5

S7. Measurement of Lasing spectra and Circular Polarization Characterization S-5

Figure S1. Chemical structure of chiral dopants S5011 and R5011..S-6

Figure S2. Chemical structure of nematic liquid crystals 5CB S-6

Figure S3. Photographs of CLCs with left-handed and right-handed helical structures S-7

Figure S4. Chemical structure of three organic dyes. S-8

Figure S5. Statistical analysis of the sizes of CLCs microdroplets. S-8

Figure S6. Transmission-mode POM of CLCs microdroplets. S-9

Figure S7. Absorption spectrum of dye-doped CLCs microdroplets S-10

Figure S8. Schematic diagram of the optical path of micro-photoluminescence (μ-PL) system. S-11

Figure S9. Lasing spectra from a series of microdroplets with different diameters............ S-12

Figure S10. Schematic illustration for the evolution of light polarization during circular polarization measurements S-13

Figure S11. Schematic diagram of chiral coating-integrated CP laser amplifying the asymmetry factor of CP lasing S-13

Figure S12. Cross-sectional SEM image of chiral coating-integrated CP laser S-14

Figure S13. POM image of chiral coating. S-14

Figure S14. Transmittance spectrum of the right-handed chiral coating S-15

Figure S15. Comparison of RCP lasing intensity with and without a chiral coating. S-15

Figure S16. Comparison of g_{lum} value. S-16

Figure S17. Circular polarization characterization of three spots (microdroplets) across the same device and on three independently prepared devices. S-17

Figure S18. Lasing emission intensity as a function of the polarization angle from the flexible CP laser device during mechanical deformation. S-17

Figure S19. Photograph of an electrically tuned CP laser. S-18

Table S1. An overview of CP lasers reported recently. S-19

Table S2. The performance parameters across three different batches of devices. S-20

References S-21

S1. Materials

1,4-bis(2-methylstyryl)benzene (Bis-MSB, >99%), Coumarin 540A (C-540A, >99%), and Nile Red (NR, >98.0%) were purchased from TCI Co.,Ltd. Nematic liquid crystals 5CB were purchased from Shanghai Aladdin Biochemical Technology Co., Ltd. Chiral dopants S5011 (99%) and R5011 (99%) were purchased from Shijiazhuang Yesheng Chemical Technology Co., Ltd. 1,4-Bis[4-(3-acryloyloxypropoxy)benzoyloxy]-2-methylbenzene (RM257, 98%), 2,2′-(ethylenedioxy)diethanethiol (EDDET, 95%), chiral monomer LC756, Diphenylamine (DPA, 99%), 2,6-di-tert-butyl-4-methylphenol (BHT, 99%), 3-(trimethoxysilyl)propyl methacrylate (TMSPMA, 97%) and 2,2-dimethoxy-2-phenylacetophenone (I-651, 99%), were purchased from Shanghai Macklin Biochemical Technology Co., Ltd. Polydimethylsiloxane (PDMS) was purchased from Dow Corning. All reagents were used as received without further purification.

S2. Fabrication of CP Lasers

First, the nematic liquid crystal 5CB (n_o=1.53, n_e=1.71 at 589 nm) was mixed with different contents of chiral dopant (S5011 or R5011) to obtain cholesteric liquid crystals with different photonic band gaps, which exhibit blue, green, and red structural color, respectively. Then, Bis-MSB (blue gain dye), C-540A (green gain dye), and NR (red gain dye) were doped into CLCs with tailored photonic band gaps. Next, the dye-doped CLCs were subjected to ultrasonic treatment for 10 minutes to achieve homogeneous dispersion. Subsequently, homogeneously dispersed dye-doped CLCs were mixed with PDMS (10:1 mass ratio of liquid silicon base and curing agent, Refractive index after solidification: 1.42) by mechanical stirring and spontaneously formed smooth spherical microdroplets of sizes in the range of 10 μm−150 μm. Finally, dye-doped CLCs microdroplets/PDMS mixtures were poured into a petri dish, and cured at room temperature for 48 hours under a nitrogen atmosphere to obtain flexible CP lasers.

S3. Fabrication of Chiral Coating-Integrated CP Lasers

The chiral coating was prepared as follows[1]: Diacrylate mesogen (RM257, 500 mg), chiral monomer (LC756, 26 mg), chain extender (EDDET, 25 mg), thermal polymerization inhibitor (BHT, 1 mg), and photo initiator (I-651, 2mg) were dissolved in toluene (425 mL). Subsequently, 50 mL of DPA solution (1:50 in toluene) was added to initiate the Michael addition reaction. The solution mixture was stirred for 20 min at room temperature for further spin coating. Then, the surface of the CP laser device was silanized with TMSPMA to ensure covalent bonding between the PDMS elastomer and chiral coating during photopolymerization. Subsequently, the solution mixture (100 μL) was spin-coated onto the CP laser device (1 cm×2 cm) at 3,000 rpm for 35 s and placed in a fume hood for 24 h to evaporate the residual toluene, followed by photocured under UV light (365 nm, 20 mW/cm^2, 5 min) to achieve robust adhesion.

S4. Fabrication of Electrically Tunable CP Laser Devices

First, the CLCs microdroplets/PDMS mixtures (20 μL) as active layer were dropped on a quartz substrate (1 cm×2 cm) coated with an indium-tin-oxide (ITO) layer, and a second piece of ITO-covered quartz glass was subsequently placed on top of the active layer. Prior to measurements, the samples were stored for 48 hours at room temperature under a nitrogen atmosphere so that the PDMS could completely crosslink. The applied electric field was delivered by a direct current (DC) power source (DINGCE, DC1533D) through the ITO electrodes.

S5. Measurement of Steady-state Photoluminescence (PL), Transmission spectra, and Circular Dichroism (CD) spectrum.

Steady-state photoluminescence (PL) measurements were performed using a custom-built optical system. A helium-cadmium (He-Cd) laser served as the excitation source at a wavelength of 325 nm. The emission signal of the sample was dispersed by a

monochromator and detected with a photomultiplier tube in conjunction with lock-in amplification. Transmittance spectra of chiral coating were recorded from 400 to 800 nm by using a Jasco V770 spectrophotometer, while the incident light angle was set at 90°, perpendicular to the sample. The circular polarized filters were positioned in front of the sample to produce left- or right-handed incident light, and the light signal transmitted through the sample was collected. The circular dichroism (CD) spectra were collected on a Chirascan spectrometer (Applied Photophysics), and the wavelength range was set from 300 to 1000 nm.

S6. Scanning Electron Microscope (SEM) Characterization and Polarized Optical Microscope (POM) Measurements

The cross-sectional SEM image of the chiral coating-integrated CP laser was observed using a Carl Zeiss Zigma Field-Emission Scanning Electron Microscope at an accelerating voltage of 5 kV. A thin conductive gold coating was applied to samples by sputter deposition before observation. POM images of CLCs microdroplets were captured using a Sdptop CX40P Polarized Optical Microscope in reflection mode.

S7. Measurement of Lasing spectra and Circular Polarization Characterization

Lasing spectra were acquired using a nanosecond pulsed laser (λ = 355 nm, pulse width = 5 ns, repetition rate = 20 Hz). Pump-intensity-dependent emission was collected with a fiber-coupled spectrometer and detected by a silicon charge-coupled device (CCD). A USB 3.0 CMOS microscopy camera (Model: YS2000) was used to capture spot size images, facilitating the calculation of spot area and subsequent determination of pump fluence from the measured intensities. To characterize the circular polarization properties of lasing emissions, a quarter-waveplate and a linear polarizer were placed in front of the fiber-coupled spectrometer, and the light signal that passed through the quarter-waveplate was collected by rotating the polarizer.

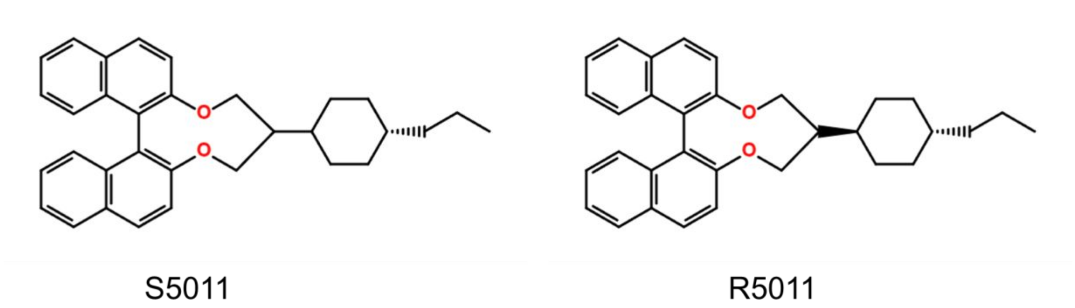

Figure S1. Chemical structure of chiral dopants S5011 and R5011. The S5011 and R5011 chiral dopants have a large helical twisting power (HTP=114 μm^{-1}) for inducing nematic liquid crystals to form CLCs with helical structure.

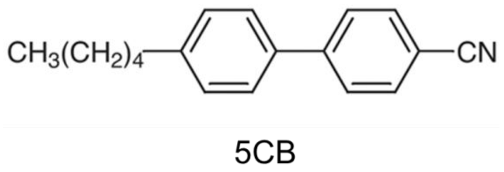

Figure S2. Chemical structure of nematic liquid crystals 5CB. CLCs were fabricated by doping different chiral dopants into the nematic liquid crystal 5CB.

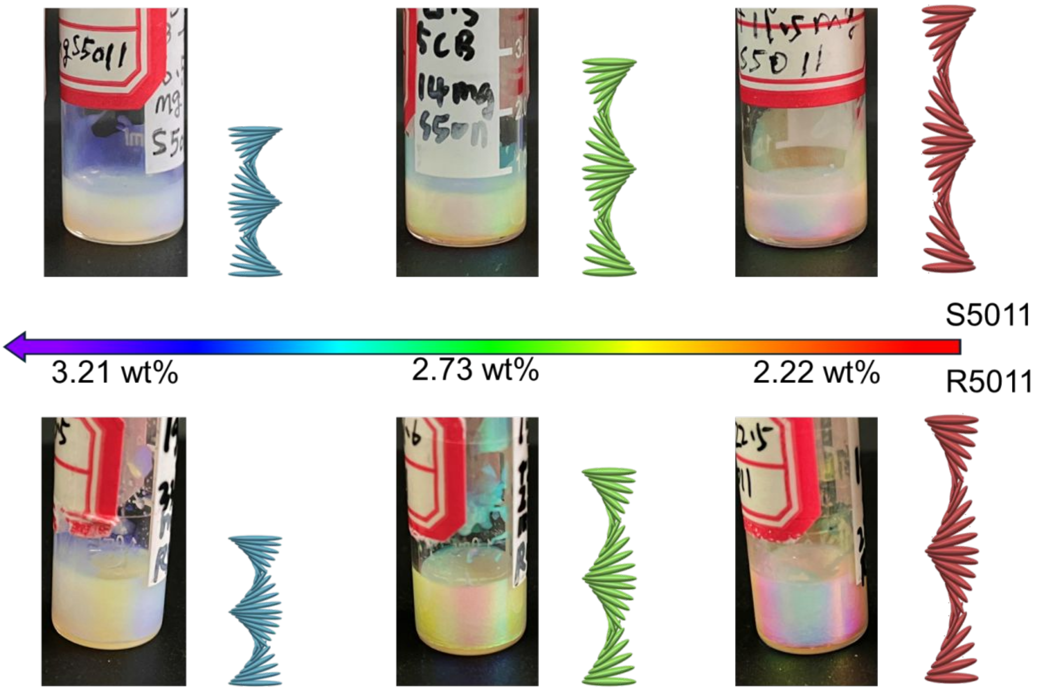

Figure S3. Photographs of CLCs with left-handed and right-handed helical structures. The chirality of CLCs was determined by the doped chiral dopants, and the photonic band gap (PBG) was adjusted by controlling the content of chiral dopants, which exhibit blue, green, and red structural colors.

The PBG of CLCs is attributed to their helical superstructures. [2] The PBG band wavelength λ of the CLCs can be determined by the following equation:

$$\lambda = \bar{n} \times p$$

where p is the pitch of the helical structure, $\bar{n}$ is the average refractive index. The effective helical pitch p can be predicted by the following equation:

$$p = \frac{1}{c \times HTP}$$

where c denotes the concentration of chiral dopant S(R)5011, HTP is the helical twisting power of the chiral dopant S(R)5011 with a value of 114 μm^{-1}.

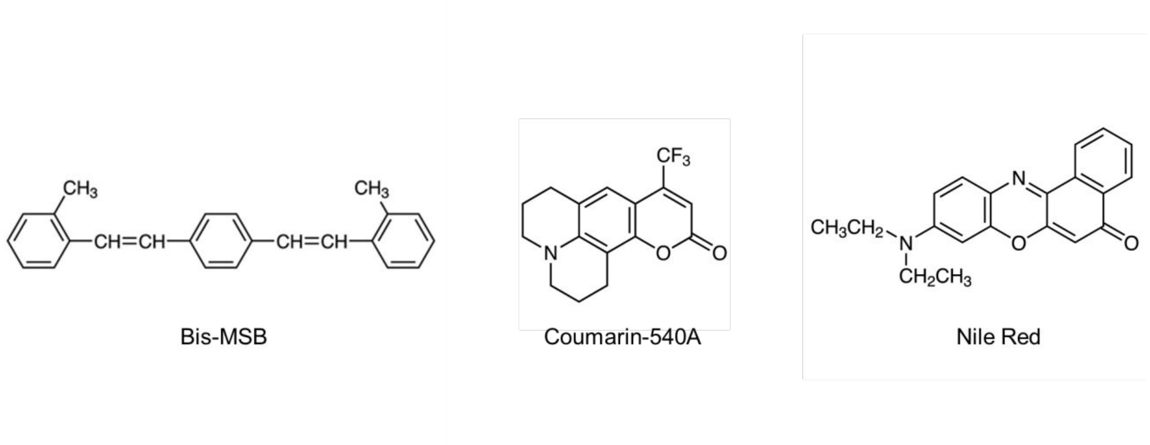

Figure S4. Chemical structure of three organic dyes. These dyes have photoluminescence (PL) emission in blue, green, and red wavebands, respectively, and were selected as gain media to dope into CLCs microdroplets for the realization of blue-, green-, and red-emissive CP lasers.

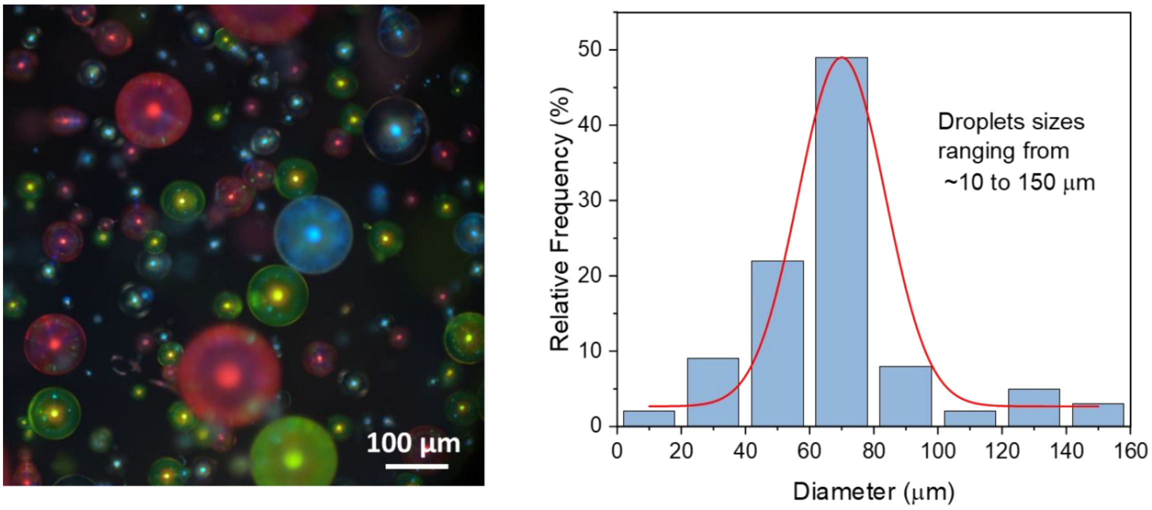

Figure S5. Statistical analysis shows the CLCs form polydisperse spherical microdroplets of sizes in the range of 10 μm−150 μm after PDMS solidification.

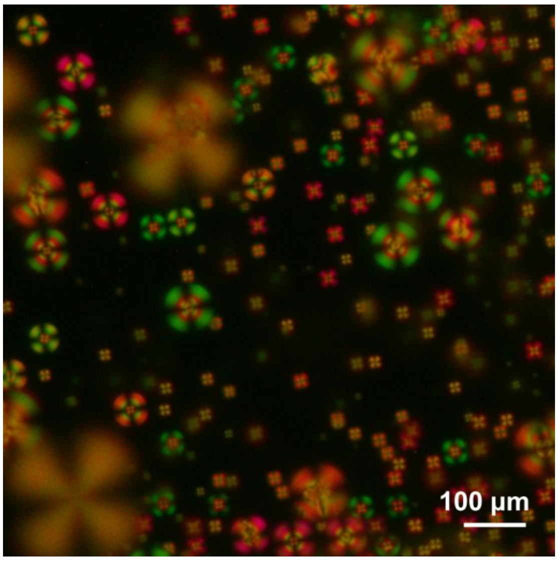

Figure S6. Transmission-mode POM of CLCs microdroplets shows the well-known Maltese cross when observed between crossed polarizers.

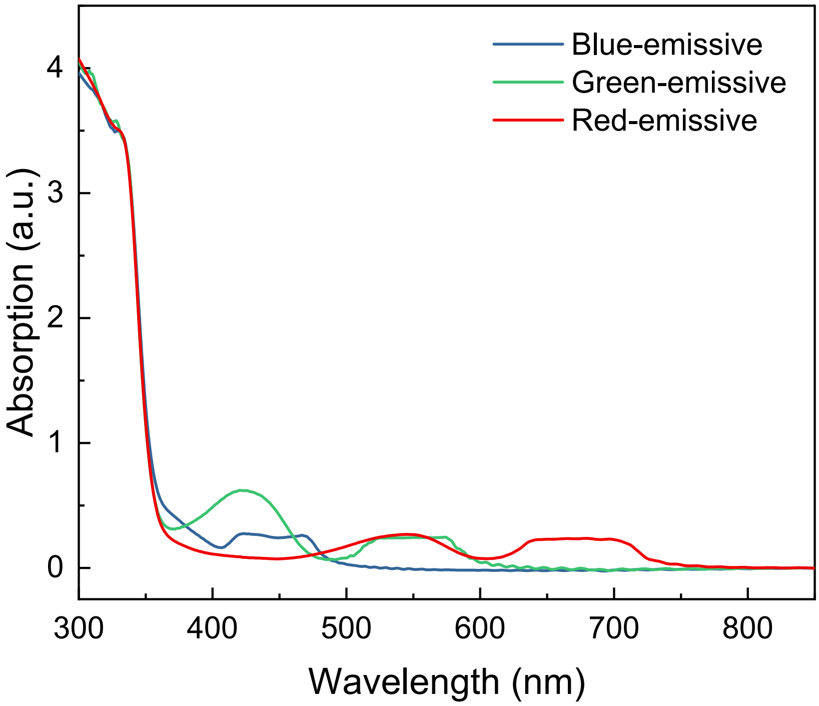

Figure S7. Absorption spectrum of dye-doped CLCs microdroplets.

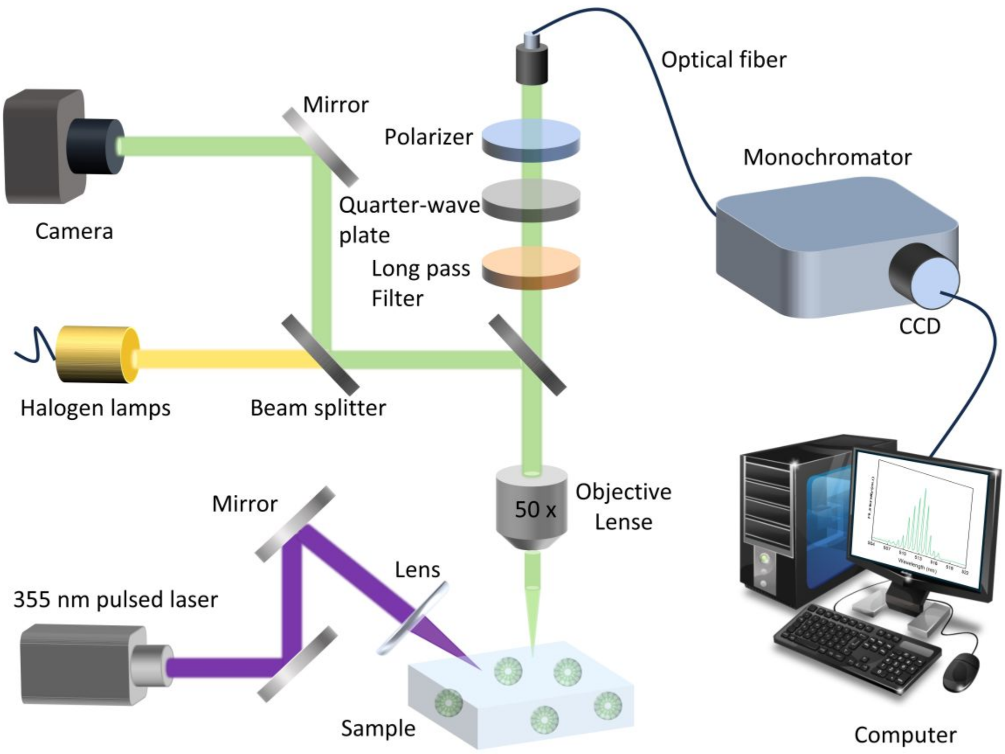

Figure S8. Schematic diagram of the optical path of micro-photoluminescence (μ-PL) system. A Q-switched Nd: YAG laser (355 nm wavelength, 20 Hz repetition rate, 5 ns pulse duration) served as the excitation source. The samples were placed on a three-axis translation stage for spatial manipulation. Emission signals were collected through a 50× objective lens in a top-view configuration with the optical output directed either to a camera for capture of optical microscopic images or to a monochromator with a silicon CCD detector for spectral analysis. Before the optical signal was delivered into the monochromator, the quarter-waveplate was placed in the optical path with a polarizer behind it.

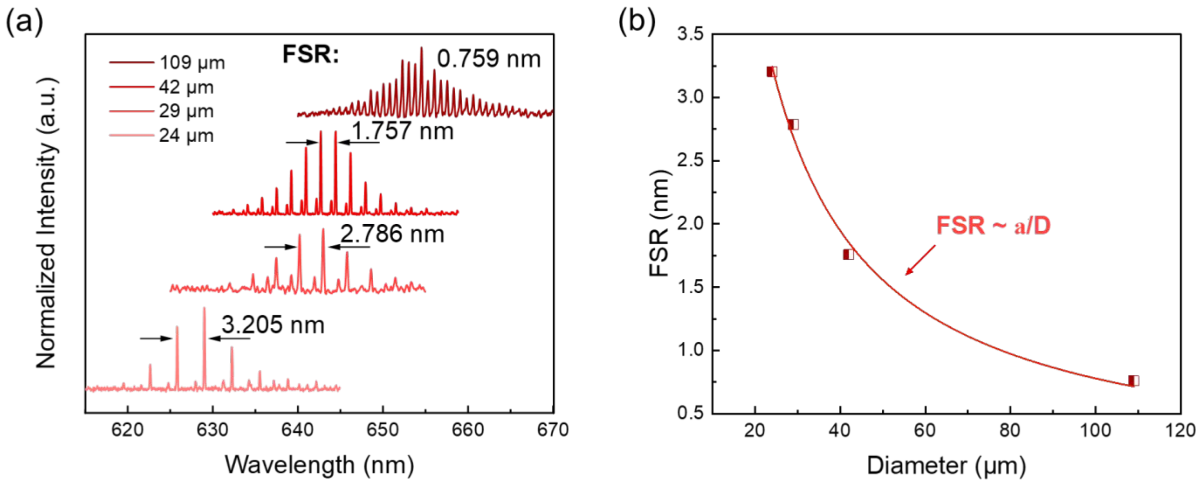

Figure S9. (a) Lasing spectra from a series of microdroplets with a diameter of 24, 29, 42, and 109 μm, respectively. (b) The FSRs as a function of the diameter of microdroplets.

The free spectral range (FSR), as a typical characteristic of WGM lasing, strongly depends on the size of a WGM cavity. [3] For the spherical WGM resonator in our work, FSR could be calculated by the equation:

$$FSR = \lambda^2 / \left(n_{eff} \pi D\right)$$

Where λ, n_{eff}, and D represent the lasing peak wavelength, effective refractive index, and the diameter of the CLCs microdroplets microcavity, respectively.

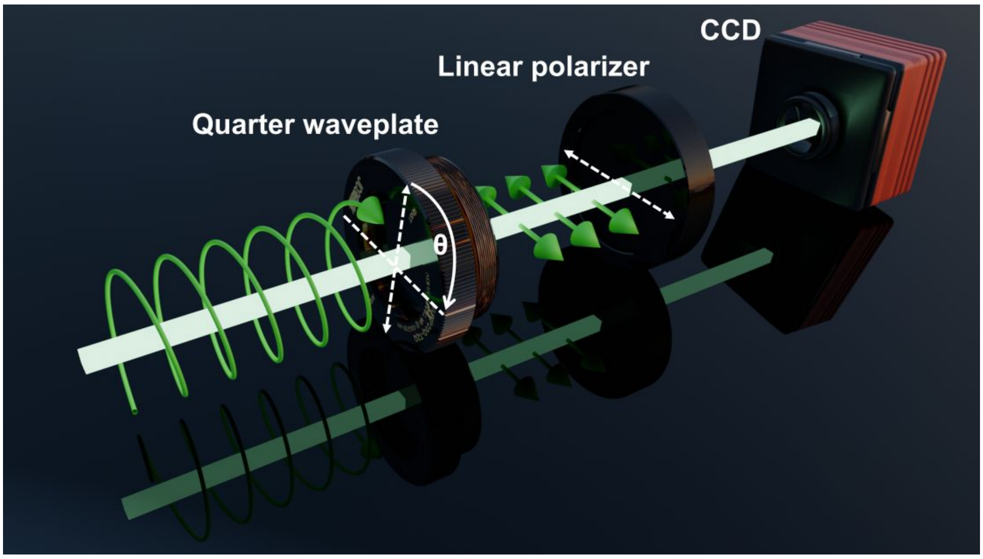

Figure S10. Schematic illustration for the evolution of light polarization. Dashed arrows stand for the long axis of the quarter-waveplate (QWP) and transmission axis of the polarizer, respectively. The QWP can convert the CP light from CP lasers into a linearly polarized light, which is detected by a rotating linear polarizer (0°−360°)

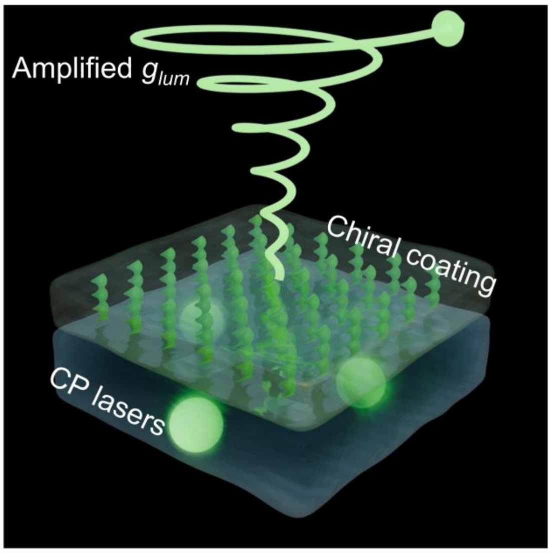

Figure S11. Schematic diagram of chiral coating-integrated CP laser amplifying the asymmetry factor of CP lasing. The chiral coating is integrated on top of the CP laser to efficiently separate CP lasing with opposite handedness to amplify the asymmetry factor for high-purity CP emission output.

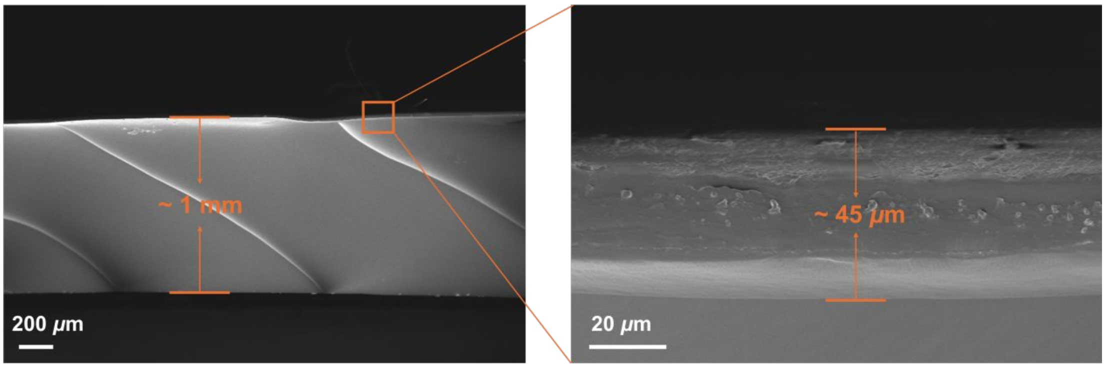

Figure S12. Cross-sectional SEM image of chiral coating-integrated CP laser.

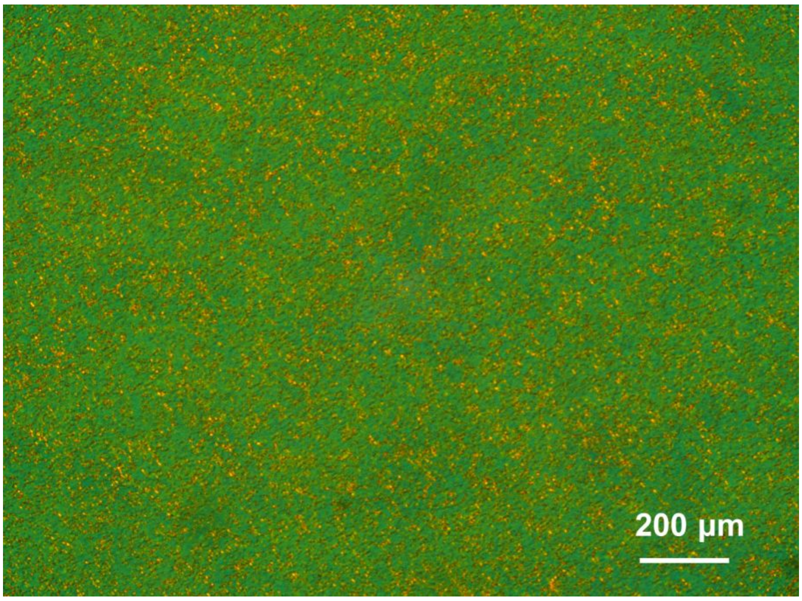

Figure S13. POM image of chiral coating.

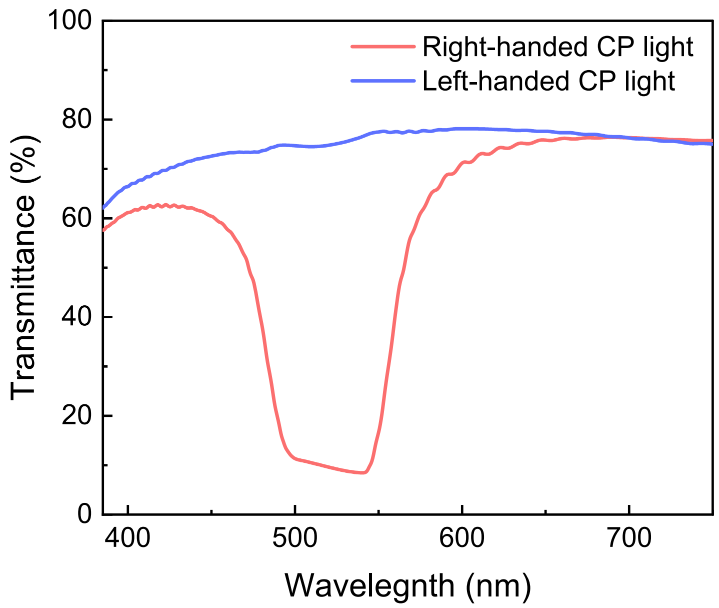

Figure S14. The transmittance spectrum of the right-handed chiral coating shows that it can suppress right-handed CP light while transmitting left-handed CP light.

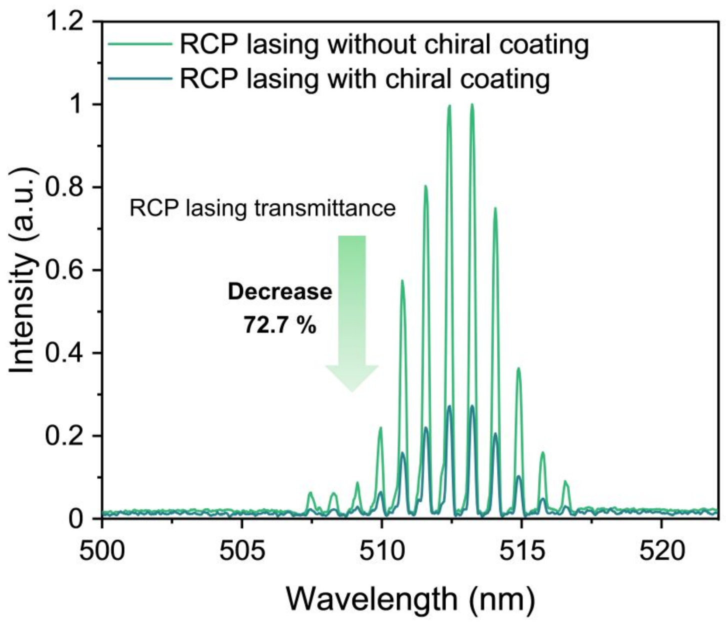

Figure S15. Comparison of RCP lasing intensity with and without a chiral coating.

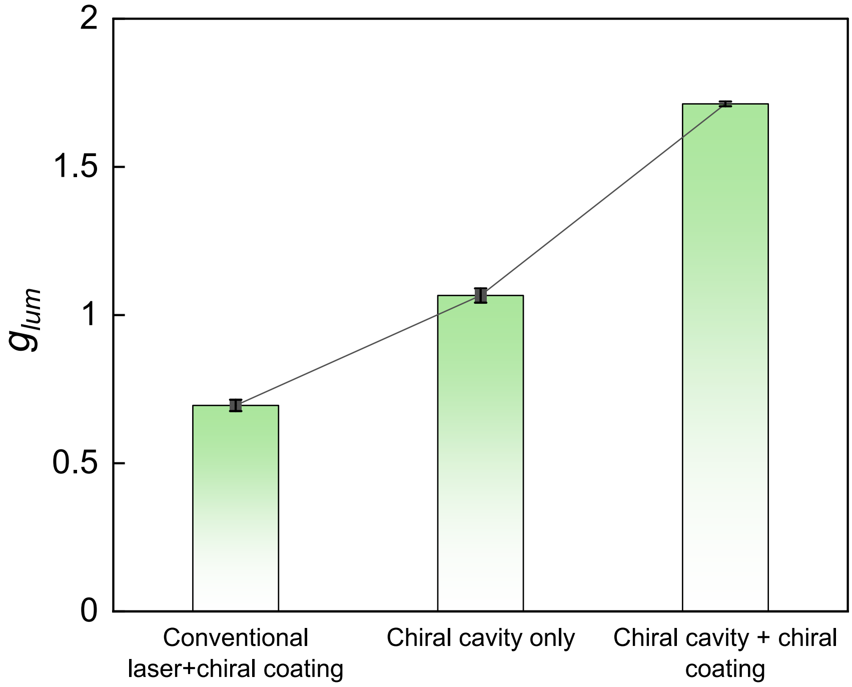

Figure S16. Comparison of g_{lum} value. In the control experiment, three devices were prepared: firstly, we used 5CB nematic liquid crystal droplets as a conventional WGM resonator (achiral cavity) to obtain a conventional laser, and then spin-coated the chiral coating. Secondly, we only use the CLCs microdroplets as chiral cavities to obtain CLCs-based CP lasers (chiral cavity only). Finally, we integrate the chiral coating onto the CLCs-based CP laser (chiral cavity + chiral coating). The results clearly demonstrate that the chiral cavity combined with chiral coating is essential for achieving the ultra-high asymmetry factor (g_{lum} = 1.72). Without the intrinsic chirality of the CLCs microdroplet cavity, the same chiral coating applied to a conventional laser only achieved a maximum glum of ~ 0.7, which is significantly lower than our reported value and also lower than the glum of the CLCs chiral cavity alone (g_{lum} = 1.06). This confirms that the chiral coating enhances the polarization selectivity already provided by the chiral cavity.

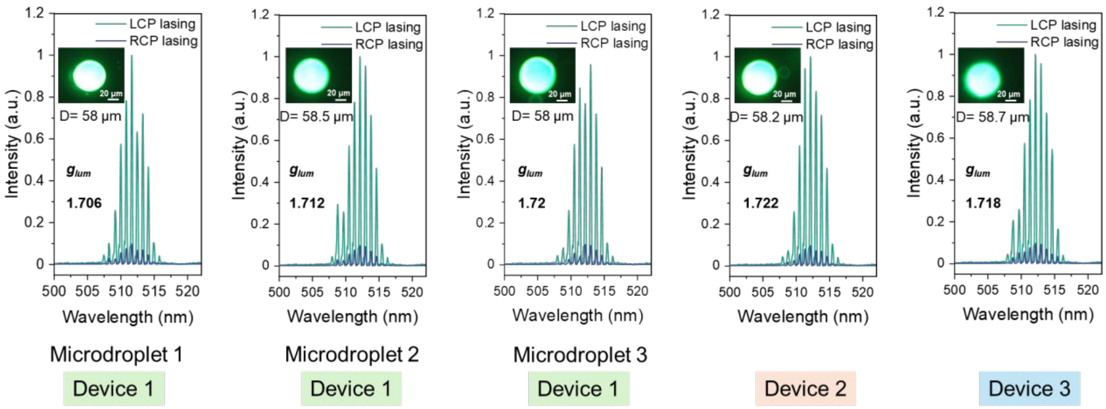

Figure S17. Circular polarization characterization of three microdroplets across the same device and on three independently prepared devices.

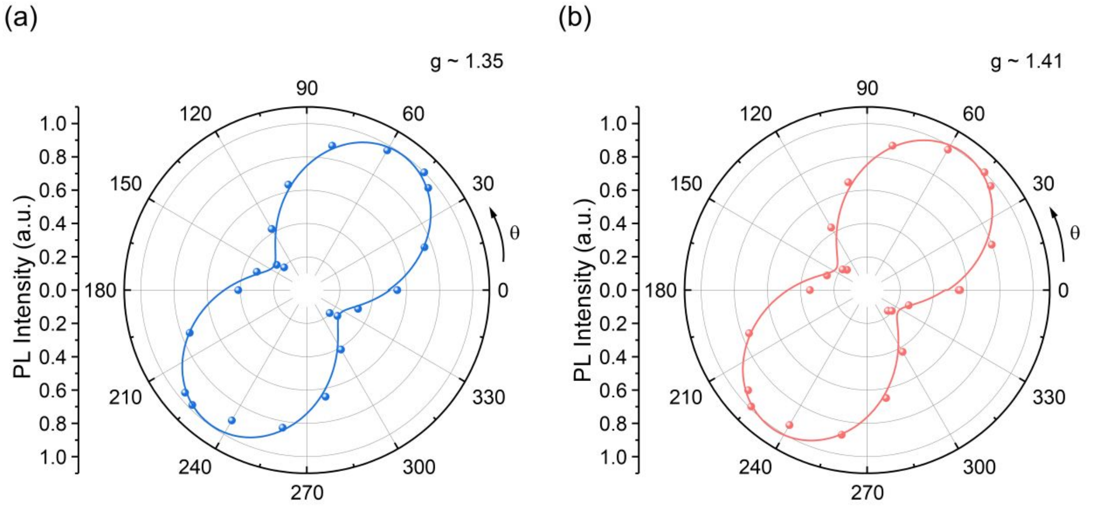

Figure S18. Lasing emission intensity as a function of the polarization angle from the flexible CP laser device during stretching (a) or bending (b) deformation (ΔL = 90 μm).

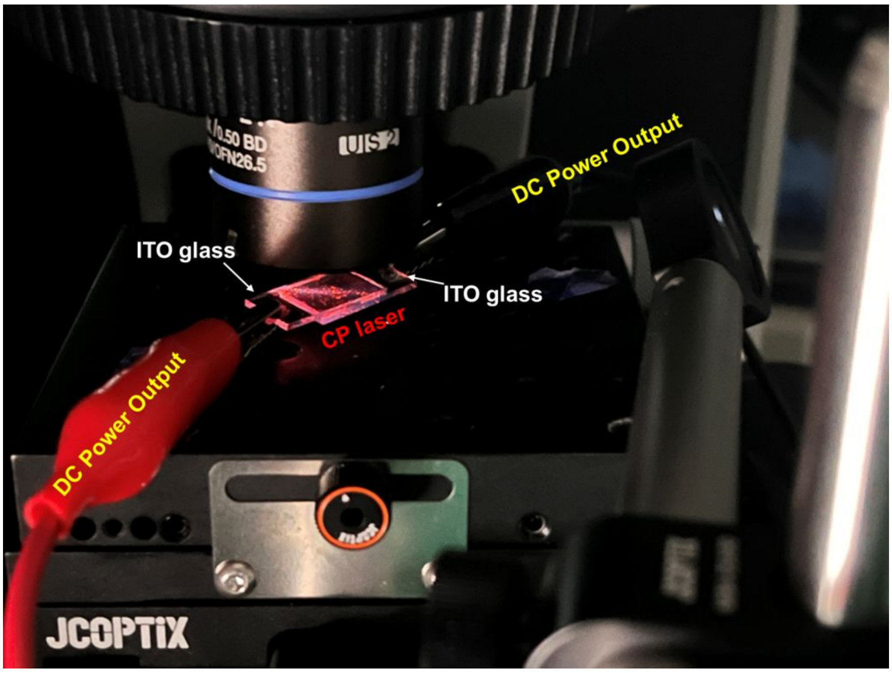

Figure S19. Photograph of an electrically tuned CP laser with a sandwich structure, which is a CP laser sandwiched between two pieces of ITO glass, with an electric field supplied through a DC power source.

Table S1. An overview of CP lasers reported recently.

Materials system[a]	Pumping Laser Type	Threshold	FWHM (nm)	Tunability	g_{lum} factor	References
Chiral AuNPs	Nanosecond	63.66 mJ cm^{-2}	—	—	0.23	(4)
Metasurface-Embedded Microcavity	Nanosecond	72 μJ mm^{-2}	—	—	0.7	(5)
Organic microcrystals	Femtosecond	25.8 μJ cm^{-2}	0.36 nm	—	1	(6)
Organic microcrystals	Femtosecond	15.31 μJ cm^{-2}	—	—	1.78	(7)
Chiral-MOFs	Nanosecond	2.3 mJ cm^{-2}	7 nm	—	0.31	(8)
CLCs-based array	Femtosecond	12.8 μJ cm^{-2}	—	—	1.6	(9)
Polymeric CLCs film	Nanosecond	—	—	Mechanically tunable	—	(10)
Polymer-stabilized CLCs	Nanosecond	1.19 μJ (Pump energy)	0.35 nm	Electrically tunable	—	(11)
CLCs-based array	Femtosecond	15.5 μJ cm^{-2}	0.7 nm	Thermally tunable	1.6	(2)
CLCs microdroplets encapsulated in PDMS elastomer	Nanosecond	**7.33 μJ cm^{-2}**	**0.09 nm**	**Dual electrically and mechanically tunable**	**1.72**	**Our work**

a) AuNPs, gold nanoparticles; MOFs, metal-organic frameworks; CLCs, cholesteric liquid crystals

Table S2. The performance parameters across three different batches of devices.

Batches of devices	Lasing threshold	Peak wavelength	g_{lum} value
Device 1 (droplets 1)	7.65 μJ cm^{-2}	507 nm ~ 516 nm	1.706
Device 1 (droplets 2)	7.76 μJ cm^{-2}	507.5 nm ~ 517 nm	1.712
Device 1 (droplets 3)	7.81 μJ cm^{-2}	507 nm ~ 516.5 nm	1.72
Device 2	7.69 μJ cm^{-2}	507 nm ~ 516.8 nm	1.722
Device 3	7.78μJ cm^{-2}	507.5 nm ~ 517 nm	1.718

Reference

(1) Li, X.; Chen, Y.; Du, C.; Liao, X.; Yang, Y.; Feng, W. Cholesteric Liquid Crystal Elastomer Coatings with Brilliant Structural Colors and Mechanochromic Response Fabricated by Spray Deposition. *Adv. Funct. Mater.* **2025**, *35* (2), 2412298.
(2) Zhan, X.; Zhou, Z.; Zhou, W.; Yan, Y.; Yao, J.; Zhao, Y. S. Wavelength-Tunable Circularly Polarized Laser Arrays for Multidimensional Information Encryption. *Adv. Opt. Mater.* **2023**, *11* (13), 2200872.
(3) Wang, Y.; Hu, Y.-H.; Wu, J.-L.; Tang, J.; Jiao, Y.-F.; Liang, Y.-C.; Wang, H.-Y.; Jiang, L.-Y.; Kuang, L.-M.; Xia, K.-Y.; et al. Microcavity-based parallel measurements of optical power and wavelength. *Appl. Phys. Rev.* **2025**, *12* (2), 021421.
(4) Lee, S.; Lim, Y.-C.; Kim, H.; Seo, D. H.; Na, J.; Kim, H.; Nam, K. T.; Jeong, Y. Random Lasing with a High Degree of Circular Dichroism by Chiral Plasmonic Gold Nanoparticles. *ACS Photonics* **2022**, *9* (2), 613-620.
(5) Yuan, Z.; Huang, S.-H.; Qiao, Z.; Wu, P. C.; Chen, Y.-C. Metasurface-tunable lasing polarizations in a microcavity. *Optica* **2023**, *10* (2), 269-278.
(6) Ren, S.; Liu, Z.-F.; Li, P.; Liu, H.; Lu, M.; Wang, K.; Yao, J.; Dong, H.; Yang, Q.-Z.; Zhao, Y. S. Circularly Polarized Lasing from Helical Superstructures of Chiral Organic Molecules. *Angew. Chem. Int. Ed.* **2025**, *64* (2), e202415092.
(7) Ji, S.; Zeng, M.; Zhan, X.; Liu, H.; Zhou, Y.; Wang, K.; Yan, Y.; Yao, J.; Zhao, Y. S. Exceptionally High-glum Circularly Polarized Lasers Empowered by Strong 2D-Chiroptical Response in a Host–Guest Supramolecular Microcrystal. *J. Am. Chem. Soc.* **2024**, *146* (32), 22583-22589.
(8) Zhu, D.; Wang, Z.; Xu, X.; Du, W.; Huang, W.; Kuai, Y.; Yu, B.; Zheng, J.; Hu, Z.; Li, S. Circularly polarized lasing from chiral metal-organic frameworks. *Photonics Res.* **2024**, *12* (8), 1654-1664.
(9) Zhan, X.; Xu, F.-F.; Zhou, Z.; Yan, Y.; Yao, J.; Zhao, Y. S. 3D Laser Displays Based on Circularly Polarized Lasing from Cholesteric Liquid Crystal Arrays. *Adv. Mater.* **2021**, *33* (37), 2104418.
(10) Shibaev, P. V.; Rivera, P.; Teter, D.; Marsico, S.; Sanzari, M.; Ramakrishnan, V.; Hanelt, E. Color changing and lasing stretchable cholesteric films. *Opt. Express* **2008**, *16* (5), 2965-2970.
(11) Lu, H.; Wei, C.; Zhang, Q.; Xu, M.; Ding, Y.; Zhang, G.; Zhu, J.; Xie, K.; Zhang, X.; Hu, Z.; et al. Wide tunable laser based on electrically regulated bandwidth broadening in polymer-stabilized cholesteric liquid crystal. *Photonics Res.* **2019**, *7* (2), 137-143.
